# Metabolic and Cardiovascular Benefits and Risks of EMD386088—A 5-HT_6_ Receptor Partial Agonist and Dopamine Transporter Inhibitor

**DOI:** 10.3389/fnins.2017.00050

**Published:** 2017-02-08

**Authors:** Magdalena Kotańska, Joanna Śniecikowska, Magdalena Jastrzębska-Więsek, Marcin Kołaczkowski, Karolina Pytka

**Affiliations:** ^1^Department of Pharmacodynamics, Faculty of Pharmacy, Jagiellonian University Medical CollegeKraków, Poland; ^2^Department of Medicinal Chemistry, Faculty of Pharmacy, Jagiellonian University Medical CollegeKraków, Poland; ^3^Department of Clinical Pharmacy, Faculty of Pharmacy, Jagiellonian University Medical CollegeKraków, Poland; ^4^Adamed Ltd.Pieńków, Poland

**Keywords:** EMD386088, 5-HT_6_ receptor partial agonist, dopamine transporter inhibitor, obesity model, anorectic activity, excessive eating model

## Abstract

Since 5-HT_6_ receptors play role in controlling feeding and satiety and dopamine is essential for normal feeding behavior, we evaluated the ability of EMD 386088—5-HT_6_ receptor partial agonist and dopamine transporter inhibitor—to reduce body weight in obese rats, as well as its anorectic properties (calorie intake reduction) in rat model of excessive eating and the influence on metabolism (plasma glucose and glycerol levels). We also determined the effect of the studied compound on pica behavior in rats and its influence on blood pressure after single administration. EMD 386088 reduced body weight in obese rats fed high-fat diet and decreased calorie intake in both models applied (rat model of obesity and of excessive eating). In both models EMD 386088 regulated plasma glucose and increased plasma glycerol levels. The latter proves that the compound reduced body fat. We think that it might have increased lipolysis, but this requires further studies. The reduction in glucose levels is the first symptom of metabolic disorders compensation. EMD 386088 did not cause pica behavior in rats but increased blood pressure after single administration. We think that partial 5-HT_6_ agonists might have potential in the treatment of obesity. Thus, EMD 386088 requires extended studies.

## Introduction

The distribution of serotonergic 5-HT_6_ receptors within the central nervous system includes hypothalamic regions, which play important role in appetite, food intake, and body weight control (Heal et al., 2008). Studies indicated that 5-HT_6_ receptor ligands might have anti-obesity activity (Woolley et al., 2001; Heal et al., 2008; Karila et al., 2015). The 5-HT_6_ receptor antagonists (i.e., PRX-07034, Ro 04-6790, BVT 5182, SB 271046) decreased food intake by enhancing satiety and caused profound weight-loss in various rodent models of obesity (Heal et al., 2008; Karila et al., 2015). Interestingly, Fisas et al. (2006) demonstrated anti-obesity effect of E-6837—a partial 5-HT_6_ receptor agonist. The compound caused significant hypophagia in non-obese and diet-induced obese rats (Fisas et al., 2006; Heal et al., 2008).

Dopamine transporter remains a potential target for antagonist or antagonist-like substitution therapies for stimulant abuse as well as obesity (Schmitt and Reith, 2010; Reith et al., 2015). Dopamine receptor agonists reduce elevated levels of the orexigenic neuropeptide and neuropeptide Y in the hypothalamus and normalize the hyperphagia, body weight gain, and hyperglycemia observed in obese ob/ob mice (Bina and Cincotta, 2000). Increased extracellular dopamine levels (caused by dopamine transporter inhibition) partially account for the hypophagic and thermogenic effects of bupropion—a dopamine and noradrenaline transporters inhibitor. Moreover, Liu et al. (2004) proved that D_1_/D_2_ receptor blockade significantly attenuated bupropion-induced thermogenesis.

Increased levels of dopamine might influence blood pressure (Volkow et al., 2003) and visceral illness (Takeda et al., 1993). Hypertension is often observed in patients treated with dopamine and noradrenaline inhibitors, such as bupropion (Cunningham and Wiviott, 2014) and tesofenasine (Appel et al., 2014). Moreover, common side effects associated with naltrexone/bupropion include nausea, vomiting (Makowski et al., 2011).

EMD386088 was first presented by Mattsson et al. (2005) as a 5-HT_6_ receptor agonist (EC_50_ = 1.0 nM) displaying selectivity over other 5-HT receptors except for 5-HT_3_. It also had no affinity for α_1_, α_2_, β_1_, D_2_, D_3_, GABA_A_, opioid μ receptors, and 5-HT transporter (Jastrzebska-Wiesek et al., 2013). Despite moderate affinity, EMD386088 acted neither as agonist nor antagonist of serotonin 5-HT_3_ receptor in biofunctional studies (Jastrzebska-Wiesek et al., 2014). Recent *in vitro* studies demonstrated that EMD386088 behaved as a potent 5-HT_6_ receptor partial agonist (Jastrzebska-Wiesek et al., 2013). EMD386088 also significantly blocked dopamine transporter (Jastrzebska-Wiesek et al., 2016).

The interesting pharmacological profile of EMD386088 indicates that such type of compound might be beneficial in the treatment of obesity. Therefore, in the present study we aimed to evaluate the influence of EMD386088 on body weight, food intake, and metabolic disturbances in high-fat diet-induced obesity model and a model of excessive eating in rats. We also determined the potential of the compound to induce emesis and its influence on blood pressure.

## Materials and methods

### Animals

The experiments were carried out on male Wistar rats. Initial body weight was: 140–160 g: obesity model, 190–220 g: model of excessive eating, or 190–220 g: rats in which the models of obesity or excessive eating were not induced. The animals were housed in pairs in plastic cages in constant temperature facilities exposed to a light-dark cycle; water and food were available *ad libitum*. Control and experimental groups consisted of six to eight animals each. All experiments were conducted according to the guidelines of the Animal Use and Care Committee of the Jagiellonian University and were approved for realization (2013 and 2015, Poland; Permissions No. 136/2013 and 258/2015).

### Drugs and compounds

**Figure 1:**
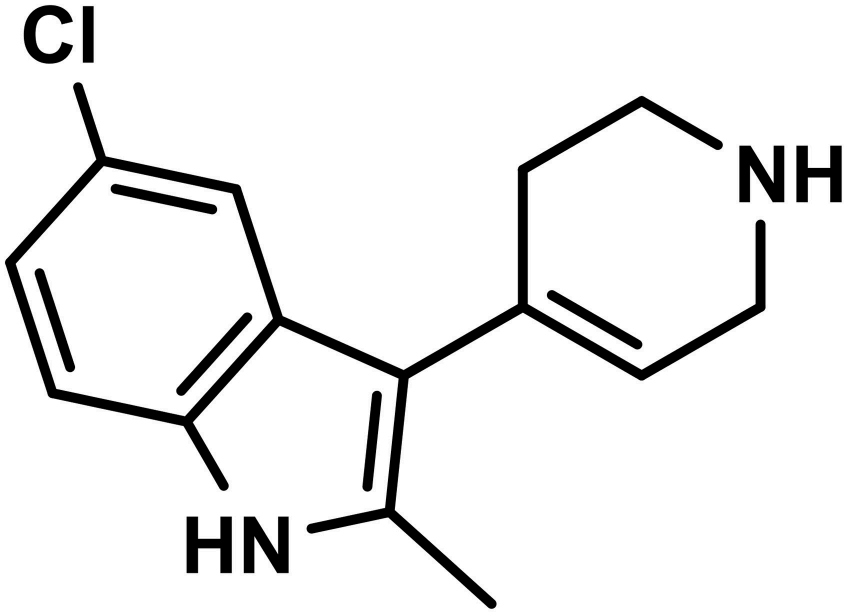
**Chemical structure of the test compound: EMD 386088**.

Heparin was delivered by Polfa Warszawa S.A. (Warsaw, Poland), while thiopental sodium and ketoprofen were from Sandoz GmbH (Austria), kethamine and xylasine from Biowet Puławy (Poland), and cefuroxime from Polfarma S.A. (Poland). EMD 386088 (Figure 1) was synthesized in the Department of Pharmaceutical Chemistry, Faculty of Pharmacy, Jagiellonian University Medical College, Kraków, Poland. We chose two doses of EMD 386088 (i.e., 2.5 and 5 mg/kg) based on our previous experiments (Jastrzebska-Wiesek et al., 2016).

### Obesity induced with a high-fat diet and influence on body weight

Male Wistar rats were pair-housed and fed high-fat diet consisting of 40% fat blend (Labofeed B with 40% lard, Morawski, Manufacturer Feed, Poland) for 14 weeks, with water available *ad libitum* (Dudek et al., 2015, 2016). Control rats were fed a standard diet (Labofeed B, Morawski Manufacturer Feed, Poland). After 10 weeks, rats with obesity induced via their diet were randomly divided into three equal groups that had the same mean body weight and were treated intraperitoneally with test compounds at the following doses: 2.5 or 5 mg/kg b.w./day and control group: vehicle—water 0.3 ml/kg (hight-fat diet + vehicle = obesity control group) once per day in the morning between 9:00 a.m. and 10:00 a.m. for 21 days. Control rats were maintained on a standard diet throughout 21 days, with intraperitoneal administration of vehicle—water (standard diet + vehicle = control group). Intakes of food and water were measured three times per week, immediately prior to administration of drugs. Animals always had free access to feed and water. On the 22nd day, 20 min after intraperitoneal administration of heparin 1000 j/rat and thiopental (70 mg/kg b.w.) plasma was collected from animals.

High-fat feed composition (932 g of dry mass): protein—193 g, fat (lard)—408 g, fiber—28.1 g, crude ash—43.6 g, calcium—9.43 g, phosphorus—5.99 g, sodium—1.76 g, sugar—76 g, magnesium—1.72 g, potassium—7.62 g, manganese—48.7 mg, iodine—0.216 mg, copper—10.8 mg, iron—125 mg, zinc—61.3 mg, cobalt—0.253 mg, selenium—0.304 mg, vitamin A—15000 units, vitamin D3—1000 units, vitamin E—95.3 mg, vitamin K3—3.0 mg, vitamin B1—8.06 mg, vitamin B2—6.47 mg, vitamin B6—10.3 mg, vitamin B12—0.051 mg, folic acid—2.05 mg, nicotinic acid—73.8 mg, pantothenic acid—19.4 mg, choline—1578 mg.

Hight-fat diet contained 100 g feed—550 kcal.

### The effect of EMD 386088 on body weight and food and water intake by non-obese rats fed palatable diet (model of excessive eating)

In order to determine the anorectic activity of EMD 386088, its effect on caloric and water intake in the model of excessive eating was assessed. Male Wistar rats (190–220 g) were housed in pair. Two groups of 6 rats were fed diets consisting of milk chocolate with nuts, cheese, salted peanuts, and 7% condensed milk and also had access to standard feed (Labofeed B, Morawski Manufacturer Feed, Poland) and water *ad libitum* for 3 weeks. Palatable control group (palatable diet + vehicle) received vehicle (water, intraperitoneally), while palatable test group (palatable diet + EMD 386088) was injected (intraperitoneally) with EMD 386088 at the dose 5 mg/kg b.w. dissolved in water. Intakes of food and water were evaluated three times per week, and body weights were measured daily immediately prior to administration of drugs. On the 22th day, 20 min after intraperitoneal administration of heparin 1000 j/rat and thiopental (70 mg/kg b.w.) plasma was collected from animals.

Palatable diet contained: 100 g peanuts—614 kcal; 100 ml condensed milk—131 kcal; 100 g milk chocolate with hazelnuts—195 kcal; 100 g cheese (Greek type)—270 kcal.

Standard diet contained 100 g feed—280 kcal.

### The effect of EMD 386088 on body weight and food and water intake by non-obese rats fed only with standard diet

Male Wistar rats (190–220 g) were housed in pair. Control group (standard diet + vehicle) received vehicle (water, intraperitoneally), while the test group (standard diet + EMD 386088) was injected (intraperitoneally) with EMD 386088 at the dose 5 mg/kg b.w. dissolved in water. Intakes of food and water were evaluated three times per week, and body weights were measured daily immediately prior to administration of drugs.

### Influence of EMD 386088 on glucose level in plasma

To determine the glucose level in plasma, standard enzymatic, and spectrophotometric tests (Biomaxima S.A. Lublin, Poland) were used. The substrate was decomposed with enzymes appropriate for the relevant product, which was converted to a colored compound. Coloration was proportional to the concentration. The absorbance was measured at a wavelength of 500 nm.

### Influence of EMD 386088 on glycerol level in diet-induced obese rats

To determine the glycerol level in the plasma, Glycerol Colorimetric Assay Kits (Cayman, USA) were used.

### Effects of EMD 386088 on visceral illness via measurement of kaolin intake (pica behavior)

To exclude the possibility that the suppression of food intake by EMD 386088 was caused by visceral illness, pica behavior was evaluated after administration. Test duration was 5 days. In addition to free access to feed, animals had free access to the white kaolin. For the first few days the animals were accustomed to the presence of kaolin in their cages. On the fourth day they were intraperitoneally given EMD 386088 at 5 mg/kg b.w. or a vehicle (negative control group) or a solution of CuSO_4_ at a dose of 6 mg/kg b.w. (1/3 LD_50_; LD_50_ = 18 mg/kg for a rat at this route of administration; positive control group). The amount of approved food, water drunk and kaolin consumed was determined after 24 h. Also, the animals were weighed prior to administration of the compound and after 24 h.

### Determination of the effect of EMD 386088 on blood pressure during 22-h study in wistar rats residing in natural housing conditions—telemetric method

The blood pressure of rats treated with EMD 386088 was measured during a 22 h period after 5 mg/kg b.w., intraperitoneally treatment with a special telemetric system—Stellar (TSE-Systems, Germany; Dudek et al., 2016).

Surgery: The operation was performed over 30 min under sterile conditions. The rats were anesthetized with kethamine and xylasine (intramuscular injection: 100 and 10 mg/kg). Before surgery and for 7 days after, animals were additionally treated with cefuroxime (20 mg/kg/day) via intramuscular injection and ketoprofen (5 mg/kg/day) via intraperitoneal injection. Blood flow in the abdominal aorta was blocked temporarily and the tip of a transmitter for measuring pressure was inserted. The transmitter was sutured to the peritoneal cavity.

The rats were individually caged for 2 weeks to heal after surgical cut. Then, the animals were placed in pairs in cages to reduce their isolation stress. Blood pressure was measured: before intraperitoneal administration of the compounds—time 0 min and 22 h thereafter.

### Statistical analysis

Statistical calculations were performed using GraphPad Prism 6 program. Results are given as arithmetic means with a standard error of the mean. Statistical significance was calculated using Student's *t*-test (if two groups were compared), one-way ANOVA with Dunnett's multiple comparison test *post-hoc* (biochemical experiments), two-way ANOVA with Bonferroni test *post-hoc* (*in vivo* experiments). Differences were considered statistically significant at: *p* ≤ 0.05.

## Results

### Influence of the test compounds on body weight and caloric and water intakes of obese rats

**Figure 2:**
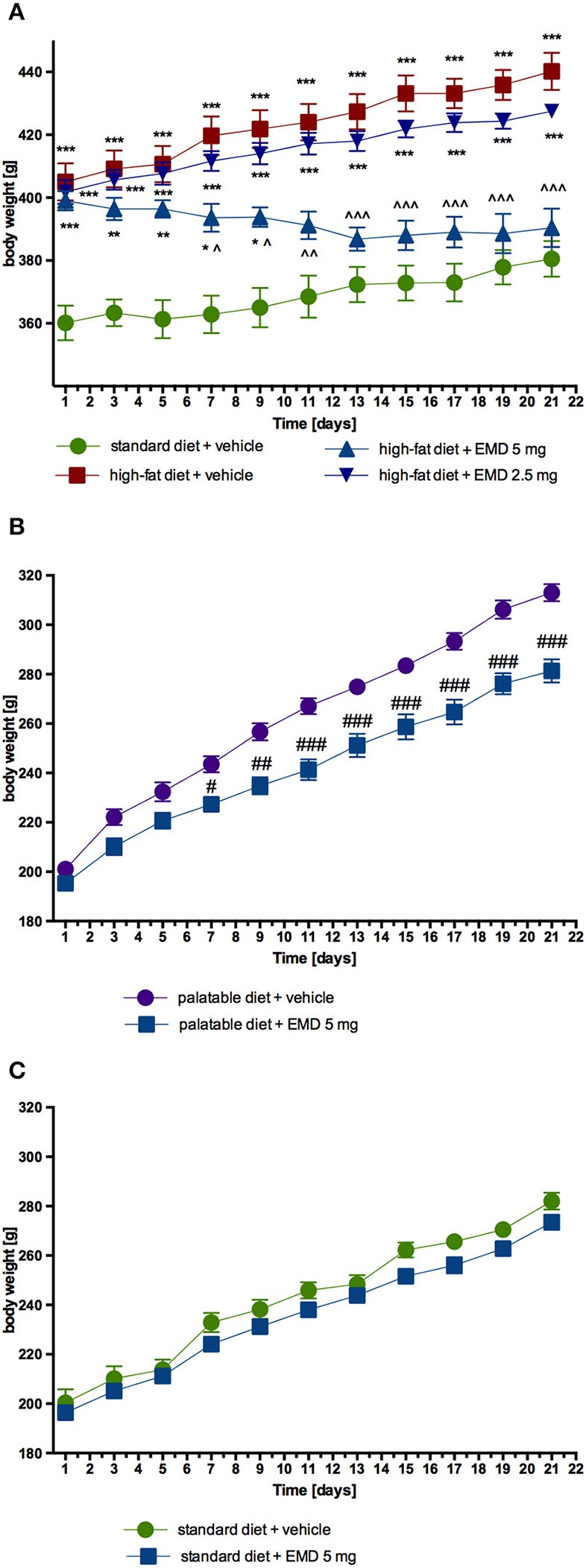
**Effect of long-term administration of EMD 386088 on body weight in male Wistar rats. (A)** The change in body weight in control (standard diet) and diet-induced obese Wistar rats, and diet-induced obese Wistar rats treated for 21 days with the tested compound; **(B)** The change in body weight in Wistar rats fed palatable diet and in Wistar rats fed palatable diet treated for 21 days with the tested compound; **(C)** The change in body weight in control (standard diet) and in Wistar rats fed standard diet treated for 21 days with the tested compound. Results are means ± SEM, *n* = 6. Multiple comparisons were performed by two-way ANOVA, Benferroni *post-hoc*. ^*^p < 0.05, ^**^p < 0.01, ^***^p < 0.001 significant vs. control rats fed standard diet; ^∧^*p* < 0.05, ^∧∧^*p* < 0.01, ^∧∧∧^*p* < 0.001 significant vs. control rats fed fat diet (diet-induced obesity control group); ^#^*p* < 0.05, ^*##*^*p* < 0.01, ^*###*^*p* < 0.001 significant vs. control rats fed palatable diet.

**Figure 3:**
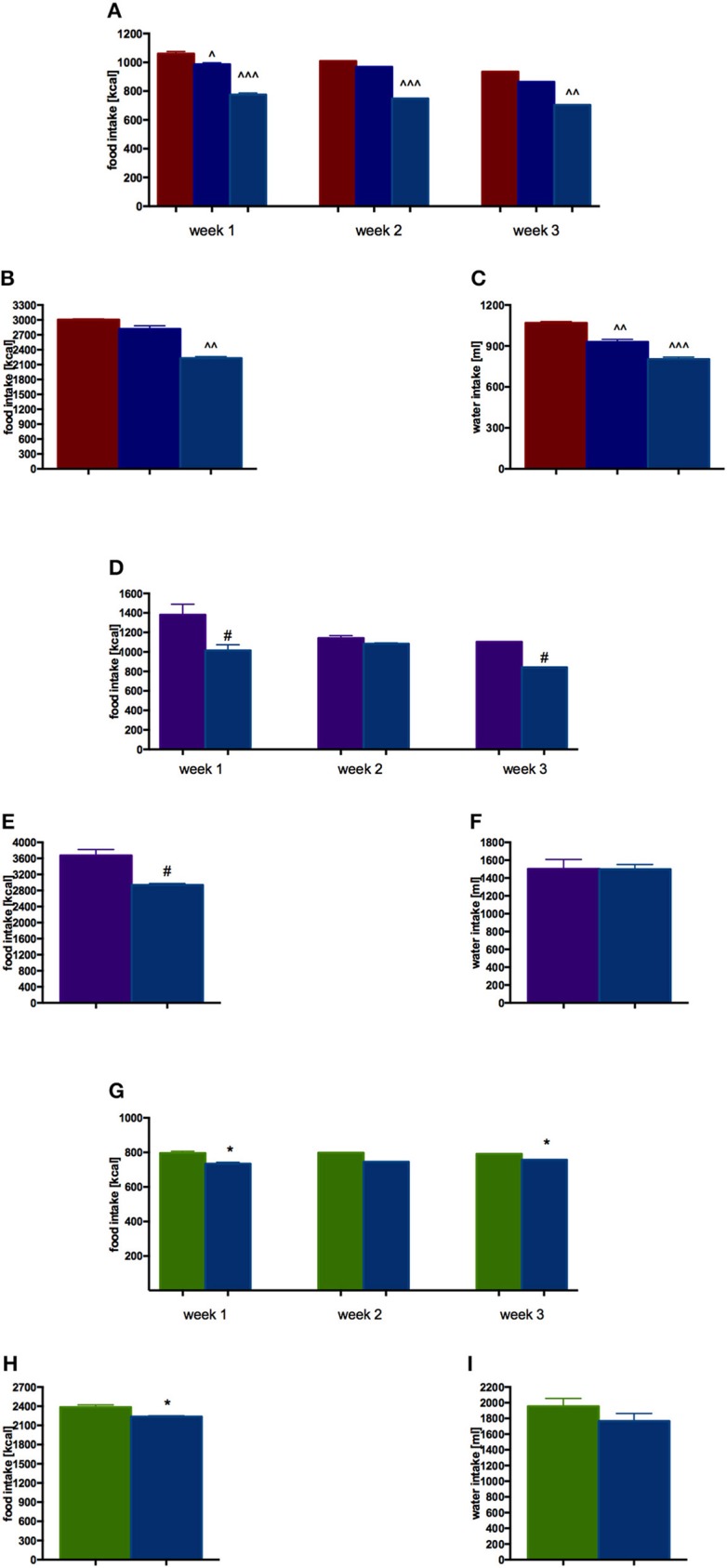
**Effect of long-term administration of EMD 386088 on food intake and water in male Wistar rats in weeks 1–3 and throughout the experiment. (A)** Obesity model—food intake in weeks 1–3; **(B)** Obesity model—food intake throughout the experiment; **(C)** Obesity model—water intake throughout the experiment; **(D)** Excessive eating model—food intake in weeks 1–3; **(E)** Excessive eating model—food intake throughout the experiment; **(F)** Excessive eating model—water intake throughout the experiment; **(G)** Standard diet fed Wistar rats—food intake in weeks 1–3; **(H)** Standard diet fed Wistar rats—food intake throughout the experiment; **(I)** Standard diet fed Wistar rats—water intake throughout the experiment. Results are means ± SEM, *n* = 4, data for two animals housed together. Multiple comparisons were performed by one-way ANOVA, Dunnett *post-hoc*, ^∧^*p* < 0.05, ^∧∧^*p* < 0.01, ^∧∧∧^*p* < 0.001 significant vs. control rats fed fat diet (diet-induced obesity control group); Comparison were performed by *t*-Student test, ^#^*p* < 0.05 significant vs. control rats fed palatable diet; ^*^p < 0.05 significant vs. control rats fed standard diet.

EMD 386088 administered intraperitoneally at a dose of 5 mg/kg b.w. to rats fed high-fat diet caused a significant decrease in body weight gain compared with the obese rats (obesity control group). The results are shown in Figure 2A. There was no significant effect in terms of reducing body weight, when the tested compound was administered intraperitoneally at a dose of 2.5 mg/kg b.w. EMD 386088 administered intraperitoneally at a dose of 5 mg/kg b.w. reduced the amount of calories consumed by the animals in the test group as compared to the obesity control group (Figures 3A,B). EMD 386088 administered intraperitoneally at doses of 2.5 and 5 mg/kg b.w. reduced the water intake in the test group as compared to the obesity control group (Figure 3C). Since the dose 2.5 mg/kg was inactive in reducing body weight and caloric intake, we used only 5 mg/kg in the following experiments.

### Effect of EMD 386088 on body weight and calorie and water intakes in model of excessive eating and in rats fed standard diet

EMD 386088 administered intraperitoneally at a dose of 5 mg/kg b.w. reduced the amount of calories consumed by the animals in the test group as compared with the control group (EMD 386088 fed palatable diet and control fed palatable diet), starting from the first week of the experiment. Results are shown in Figures 3D,E. Similar differences were observed in calorie intake in the group with access only to standard feed (EMD 386088 fed standard diet and control fed standard diet). Results are shown in Figures 3G,H.

Similar to calorie intake, animals fed palatable feed and treated with EMD 386088 showed significantly less weight gain than animals in the control group consuming a preferential feed (Figure 2B). From the seventh day of the experiment there was a statistically significant difference between the groups. The difference in weight on the seven day amounted to 5.02%, and on the twenty-first day to 16.09%. Most importantly, the body weight of rats treated with EMD 386088 and having access to preferential feed did not differ significantly from the weight of control animals fed standard feed. No effects on body weight were noted in animals treated with EMD 386088 and consuming standard feed. Results are shown in Figure 2C.

There were no significant differences in terms of the intake of water by animals in groups fed preferential feed and groups fed standard feed. Results are shown in Figures 3F,I.

### Influence of EMD 386088 on plasma glucose and glycerol levels in diet-induced obese rats

**Figure 4:**
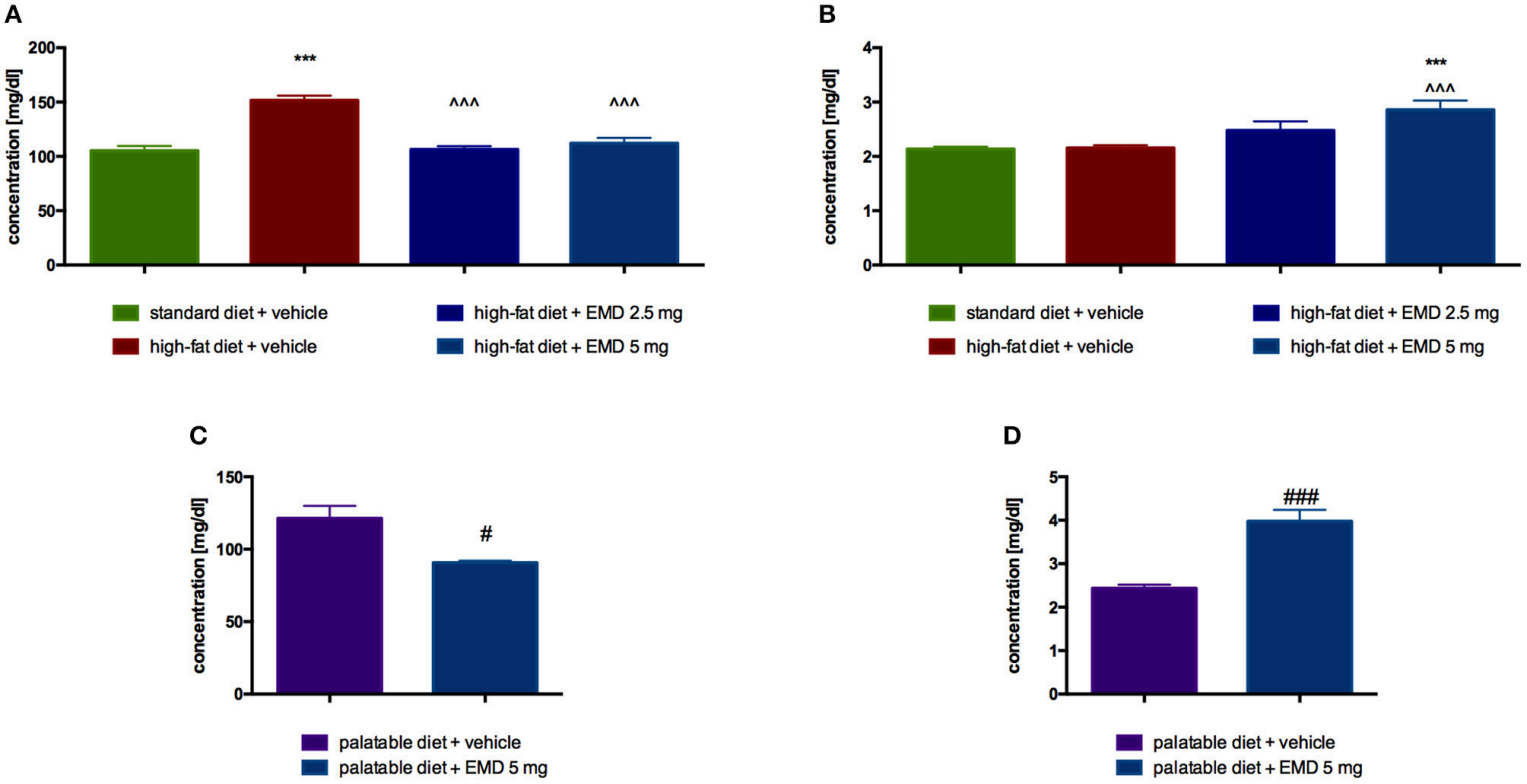
**Effects of long-term administration of EMD 386088 on plasma glucose level in male Wistar rats in diet-induced obesity model (A)** or excessive eating model **(C)** and on plasma glycerol level in male Wistar rats in diet-induced obesity model **(B)** or excessive eating model **(D)**. Results are means ± SEM, *n* = 6. Concentrations in plasma: mg/dl. Comparisons vs. the vehicle-treated control group (^*^) or vs. the vehicle-treated obesity (∧) or excessive eating control groups (#) were performed by Student's *t*-test (if two groups were compared) or one-way ANOVA, Dunnett *post-hoc*. Significant differences are denoted by ^***^p < 0.001, ^∧∧∧^*p* < 0.001, ^#^*p* < 0.05, ^*###*^*p* < 0.001.

In animals treated intraperitoneally with the tested compound at a dose of 5 mg/kg b.w., significantly lower blood glucose was observed as compared with a group of obese animals treated with water. Results are showed in Figure 4A. EMD 386088, at a dose of 5 mg/kg b.w., significantly increased plasma glycerol levels in rats fed a high-fat diet, in comparison with the level of glycerol in the obese control group (Figure 4B).

### Influence of EMD 386088 on plasma glucose and glycerol levels in the model of excessive eating

Glucose level was significantly lower in the group treated with the tested compound at a dose of 5 mg/kg b.w., compared with the level determined in the plasma of the control group fed palatable feed (Figure 4C). EMD 386088, at a dose of 5 mg/kg b.w., significantly increased plasma glycerol levels in rats fed palatable feed, in comparison with the level of glycerol in the control group. The results are presented in Figure 4D.

### Effects of EMD 386088 on visceral illness via measurement of kaolin intake (pica behavior)

**Figure 5:**
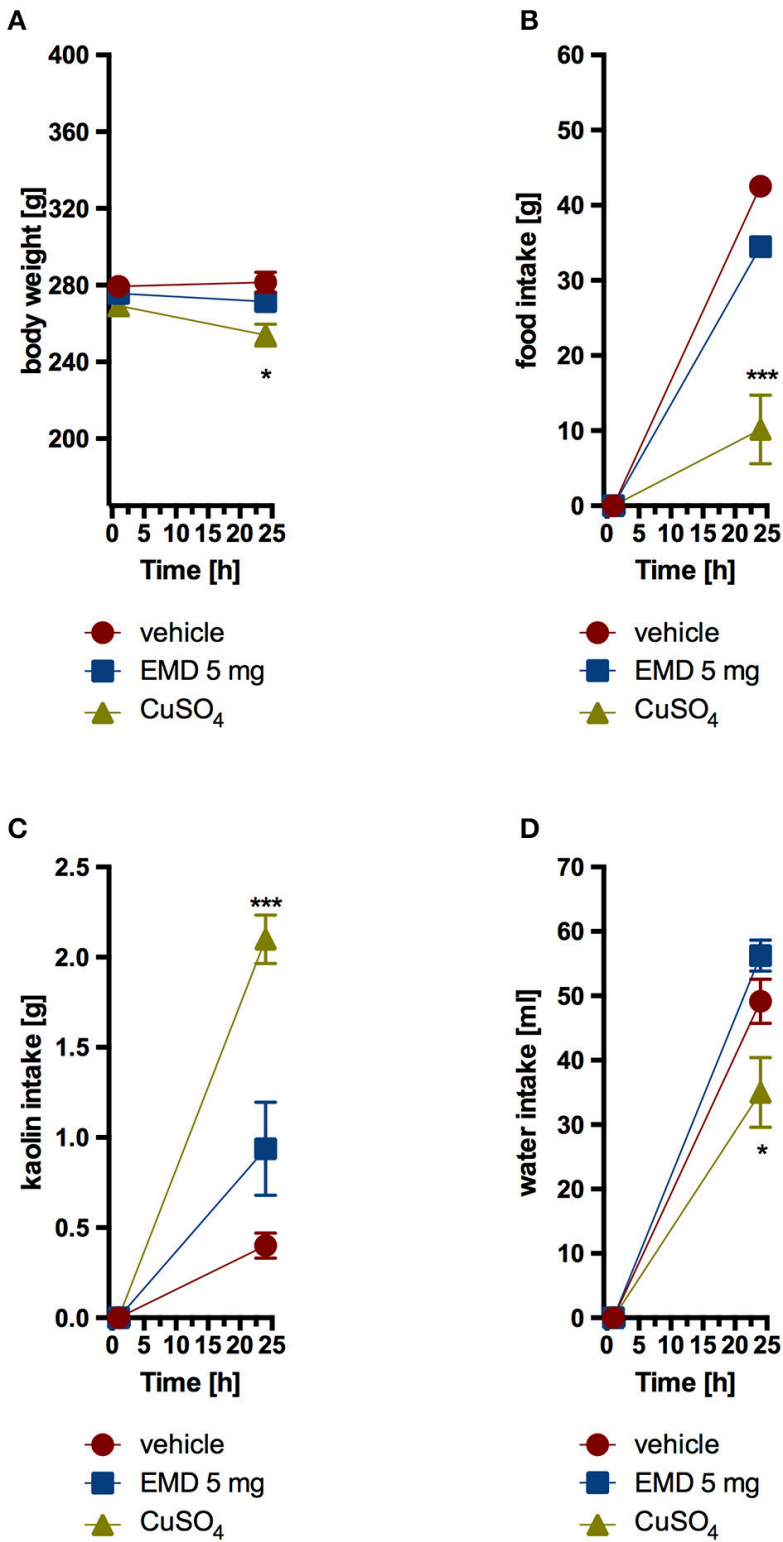
**Effect of one administration of EMD 386088 on body weight (A)**, food intake **(B)**, kaolin intake **(C)**, and water intake **(D)** in male Wistar rats in the model of pica behavior. Results are means ± SEM, data for two animals housed together, *n* = 6. Multiple comparisons against the vehicle-treated control group (^*^) were by two-way ANOVA, Bonferroni *post-hoc*. Significant differences are denoted by ^*^p < 0.05, ^***^p < 0.001.

Animals that received EMD 386088 intraperitoneally at a dose of 5 mg/kg b.w. did not consume more kaolin compared with the control group which received only vehicle. Also, a comparable amount of feed and water was ingested. The second control group, which was given CuSO_4_, had a significantly higher kaolin intake and a significantly lower feed and water intake. Results are shown in Figure 5.

### Influence of EMD 386088 on blood pressure—telemetric method

**Figure 6:**
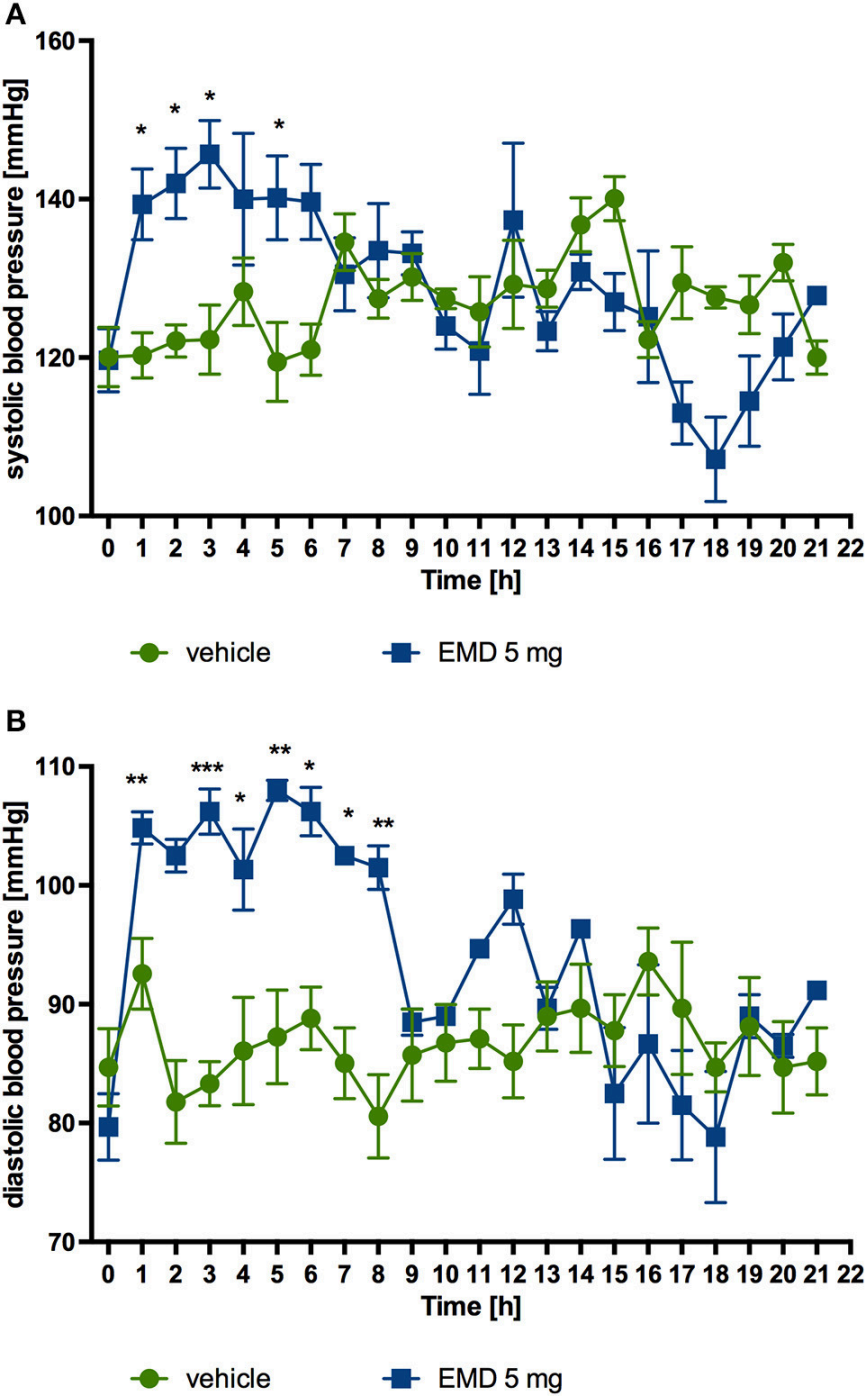
**Changes in the blood pressure after single administration of EMD 386088 to normotensive rats (telemetric method)**. Changes in systolic **(A)** and diastolic **(B)** blood pressure after single, intraperitoneal administration of the test compound at a dose of 5 mg/kg b.w. to rats fed standard diet. Mean ± SEM; n = 6 (two-way ANOVA, Bonferroni post-hoc). ^*^p < 0.05, ^**^p < 0.01, ^***^p < 0.001 significant vs. control rats.

After single intraperitoneal administration of EMD 386088 at a dose of 5 mg/kg b.w. to rats residing in natural housing conditions, there was a significant rise in systolic and diastolic pressure to the 5th and 8th h after administration, respectively, compared with rats that received water. Figures 6A,B show the results.

## Discussion

We found that EMD 386088—a partial 5-HT_6_ agonist and dopamine transporter inhibitor—reduced body weight and decreased calorie intake in obese rats and in the rat model of excessive eating. In both models EMD 386088 regulated plasma glucose and increased plasma glycerol levels. The compound did not cause emesis after single administration, but it increased blood pressure.

Mattsson et al. (2005) introduced EMD 386088 as selective (except for 5-HT_3_), high-affinity 5-HT_6_ receptor agonist. However, our previous studies proved that the compound behaved as partial 5-HT_6_ agonist (Jastrzebska-Wiesek et al., 2013). Moreover, EMD386088 possessed significant affinity for dopamine transporter (Jastrzebska-Wiesek et al., 2016). Despite the affinity for serotonin 5-HT_3_ receptors, we showed that EMD 386088 acted neither as agonist nor antagonist in biofunctional studies (Jastrzebska-Wiesek et al., 2014). *In vivo* experiments showed antidepressant- and anxiolytic-like properties of the compound in rats (Jastrzebska-Wiesek et al., 2014, 2015, 2016). Interestingly, Nikiforuk et al. (2011) demonstrated that EMD 386088 ameliorated ketamine-induced deficits relevant to schizophrenia in rats. Since 5-HT_6_ receptors play role in controlling feeding and satiety (for review see Voigt and Fink, 2015) and dopamine is essential for normal feeding behavior (Billes and Cowley, 2007), we decided to evaluate the ability of EMD 386088 to reduce body weight in obese rats, as well as its anorectic properties (calorie intake reduction) in rat model of excessive eating and the influence on metabolism. Given the significant affinity of EMD 386088 for dopamine transporter, we also investigated pica behavior in rats and the effect on blood pressure after single administration.

Our results demonstrate that EMD 386088 at the dose 5 mg/kg (but not 2.5 mg/kg) significantly decreased body weight in obese rats fed high fat diet compared with vehicle-treated obese controls. Since 5-HT_6_ receptor ligands influence satiety center, we evaluated if EMD 386088 reduced palatable food consumption in normal rats (model of excessive eating). The experiments revealed that rats treated with EMD 386088 (5 mg/kg) consumed less palatable food than vehicle-treated controls. Moreover, we demonstrated that EMD 386088 administered to normal rats had no effect on body weight. Weight disorders in animals with normal body weight could be described as an unacceptable side effect, but this effect did not occur. We also showed that EMD 386088 normalized plasma glucose levels and increased plasma glycerol in both models applied. The latter proves that the compound reduced body fat. We think that it might have increased lipolysis but this issue requires further studies. The reduction in glucose levels is the first symptom of metabolic disorders compensation. Our results are consistent with the results obtained by Fisas et al. (2006), who demonstrated anti-obesity effect of E-6837—a partial 5-HT_6_ receptor agonist.

As mentioned earlier the data concerning the effect of 5-HT_6_ receptor ligands is ambiguous. Nevertheless, according to some authors 5-HT_6_ receptor antagonists reduce inhibitory effects of GABA on pro-opiomelanocortin in the arcuate nucleus neuron and consequently inhibit hunger signal induction (Sargent and Henderson, 2011). Since similar to antagonists, partial agonists reduce the response of full agonists (but to a lesser extent), we think that this might be EMD 386088 mechanism of action. Moreover, studies demonstrated that dopaminergic pathways play pivotal role in food consumption regulation (Baik, 2013). Scientists proved that compared with non-obese controls obese patients showed reduced dopamine D_2_ receptor availability or activity (Wang et al., 2001; Stice et al., 2008; Volkow et al., 2008). Volkow et al. (2002) showed that palatable food was associated with dopamine release in healthy subjects. Conversely, in obese patients the response to palatable food was reduced (Stice et al., 2010). Altogether, the data proves that drugs rebalancing dopamine system might be beneficial in the treatment of obesity. Accordingly, Appel et al. (2014) showed that tesofenasine, a compound which inhibited, among others, dopamine re-uptake, reduced body weight. Therefore, we think that dopamine transporter inhibition contributes to EMD 386088 anti-obesity effects.

5-HT_3_ receptor agonists may influence behavioral feeding, have anorectic properties, and reduce body weight (Voigt and Fink, 2015; e.g., SR-57227 Li et al., 2015). As mentioned above, EMD 386088 had moderate affinity for serotonin 5-HT_3_ receptor, but our biofunctional studies showed, that it acted neither as agonist nor antagonist of serotonin 5-HT_3_ receptor (Jastrzebska-Wiesek et al., 2014). Therefore, we did not consider the interaction with 5-HT_3_ receptor as potential mechanism of anorectic and body-reducing action of EMD 386088.

Since EMD 386088 blocked dopamine transporter and increased dopamine levels might cause emesis (D_2_ receptors activation in the chemoreceptor trigger zone), we evaluated pica behavior (kaolin intake) in rats. Pica behavior in rats is analogous to vomiting in other species and is mediated by the same mechanisms as in humans (Takeda et al., 1993). We showed that single administration of the studied compound did not influence kaolin intake in rats. Thus, we can exclude the possibility that the suppression of food intake was due to visceral illness.

Since the studied compound inhibited dopamine reuptake, we determined the effect of single administration of EMD 386088 on blood pressure in rats (preliminary studies). The compound increased systolic blood pressure for the first 5 h and diastolic for 8 h after injection. After that time blood pressure decreased to the baseline values. We think that this temporal increase in blood pressure was due to dopamine transporter inhibition and consequent increase in dopamine level. Currently used anti-obese drugs, such as bupropion, increase dopamine levels, and thus increase blood pressure (Makowski et al., 2011; Cunningham and Wiviott, 2014). Body weight reduction improves metabolic disorders and decreases cardiovascular disease risk factor (Rosin, 2007; Soran et al., 2016). The increase in blood pressure would be undesirable in patients with essential hypertension and/or arrhythmias, in whom cardiovascular disorders are not associated with obesity. In these patients, dopamine transporter inhibitors should be avoided or used very carefully under medical supervision. We should emphasize that very often a tolerance develops to the temporal increase in blood pressure after catecholamine reuptake inhibitors (Makowski et al., 2011). Moreover, body weight reduction and the metabolic disorders improvement contributes to the reduction of hypertension coexisting with obesity. Therefore, in case of the long-term use of such drugs, the initial effect on blood pressure is not so important.

The limitation of our study was that the blood pressure was not evaluated throughout the experiment (for 21 days). Therefore, in future studies we plan to confirm our findings using other models of obesity (e.g., 30% fructose intake or db/db mice) and simultaneously monitor blood pressure and heart rate in animals (evaluation of cardiovascular disease risk factor). Given the high affinity for dopamine transporter, we also plan to evaluate the influence of EMD 386088 on locomotor activity of rodents after chronic treatment.

## Conclusions

In conclusion we demonstrated that EMD 386088—a partial 5-HT_6_ agonist and dopamine transporter inhibitor—reduced body weight in obese rats and decreased calorie intake in rat models of obesity and excessive eating. In both models EMD 386088 regulated plasma glucose and increased plasma glycerol levels. The compound did not cause pica behavior in rats but increased blood pressure after single administration. We think that partial 5-HT_6_ agonists might have potential in the treatment of obesity. Thus, EMD 386088 requires extended studies.

## Author contributions

Conceived and designed the experiments: MKotańska. Performed the experiments: MKotańska. Analyzed the data: MKotańska, KP. Contributed reagents/materials/analysis tools: JŚ, MKołaczkowski. Wrote the paper: MKotańska, KP, MJ.

## Funding

This work was supported by statutory funds from the Faculty of Pharmacy, Jagiellonian University Medical College, Krakow, Poland (K/DSC/001953).

### Conflict of interest statement

The authors declare that the research was conducted in the absence of any commercial or financial relationships that could be construed as a potential conflict of interest.
